# Magnetic Hygiene

**Published:** 1889-03

**Authors:** S. Helen Clarke


### MAGNETIC HYGIENE.

PART III.

As the state of repose places our organism in a negative condition, when little resistance can be opposed to deleterious influences, it is evident that sleep should be obtained under the most favorable auspices in order to gain from it that recuperation which nature intends it shall give.

Sleeping-rooms should be spacious, provided with ventilators, have a moderate and even temperature, and if lighted at all, only the most subdued and mellow light should be allowed. Every individual should have the privilege to seek rest in a condition of perfect independence.

No two persons should occupy the same couch unless both are in a flourishing condition of health and vigor, of about the same age and temperament and entertaining for each other such high sympathetic regard and harmonious feeling of affection that all selfish tendencies become obliterated between them. The pernicious custom of permitting youth and old age, health and disease, vigor and infirmity, selfishness and abnegation, sympathy and antipathy to indiscriminately sleep together is the promoter of a great deal of moral torture and of much physical suffering.

As we have previously stated, the human organism is a magnet and subjected to the laws regulating magnetic action, but it is an animated magnet, and the will of the intelligent operator which animates it, the ego, directs and controls many of its movements. The quantity, quality and direction of its power to act depends also, in a measure, upon the faculties of the individual as well as upon the state of perfection of the physical structure, for the more the body becomes depleted by overwork, disease, or old age, the more it becomes powerless to adapt to its use the crude magnetic force of nature, and therefore, loses its power of transmission.

Indeed, it is the natural tendency of all such organisms to draw heavily upon and assimilate to their use the vital powers of more vigorous and healthy individuals with whom they may be brought in contact. This being a natural tendency it proceeds independently of the will of those concerned, a depleted organism absorbing animal magnetic force as readily as a dry sponge absorbs water. Therefore, should a healthy and robust individual with full power of transmission, be repeatedly placed, while in the negative state, in prolonged contact with one capable of little else than absorption, it is easy to comprehend that while the latter is benefited it is to the detriment of the former. In the positive or wakeful state our will power can, to a great extent, stem the current and prevent transmission ; on the other hand, it can increase it if it chooses to act in co-operation with the natural tendency.

Transmitted in this wise, animal magnetism becomes a powerfully recuperative and vitalizing agent, capable of reaching to the very centre of life itself. But to attain this attitude it must become spiritualized, the whole process must be actuated by most unselfish feelings of humanitarian love, great sympathy and sincere aspirations to benefit.

Considered from the standpoint of hygiene we see how essential to our welfare are the knowledge and application of the laws related to magnetic action of the human body. It teaches not only how to husband our vital powers, how to rationally employ the means devised by nature for their recuperation, but also, the great worth of animal and spiritual magnetism as a restorative agent in cases of depressed mental energy, or impaired physical power.

It teaches, also, that in many instances confirmed conditions of disease yield readily to magnetic power, and that treatment through this agency *when given under the conditions required for success*, is always beneficial and never harmful.

Hence, wisdom should lead us to apply our knowledge of hygienic principles and call to our aid the beneficial influence of magnetic treatment whenever premonitory symptoms indicate that our health is threatened and before diseased conditions become fully developed.

Any one subjected to loss of vital power through great mental strain or depleting manual labor, will find in animal magnetism a restorative more natural, safer, more potent, beneficial and lasting in effect than all the stimulants and tonics of the materia medica.

If struggles, reverses, inharmony, so often encountered on this mundane sphere, render you despondent, make life a burden ; if moral torture, anguish deep and lasting, threaten even your reason, despair not, neither wait until irreparable damage is done, but, at once, seek the remedy that will not only vitalize your organism, but comfort, uphold and cheer you.

*Spiritual magnetism* evolved from thoughts of love and tenderness, from feelings of pity and sympathy, from constant aspirations to uplift and sustain, is the God-given remedy that can minister unto you, lift up the dreary pathway, console and sustain the despondent mind, soothe and heal the wounded and suffering spirit.

It is the power of good, the power of truth, the power of knowledge that with it brings new life, new strength, for it is of God. It is a subtle, spiritual essence, an inspiration from on high that, like a ray of sunlight, will invade your being, bringing brightness and cheerfulness wherever it penetrates. Dispensed knowingly and with understanding, wonders may be done with it, and for the welfare of humanity it is to be hoped that the knowledge of magnetic hygiene and magnetic therapeutics will become so generalized that through their application much unnecessary suffering may be prevented and a more natural, rational and safe method of healing than the old effete systems of blind dosing and wholesale poisoning be accepted and propagated.

S. Helen Clarke.

